# Indents

**Published:** 1901-10-15

**Authors:** 


### INDENTS.

**☞ Brief Articles of Technical or Scientific Value will receive notice and comment. Contributions invited.**

FOR SMOKERS' GINGIVITIS.—

℞ Salol, - - - - - - ʒj;

Tinct. Catechu, - - - - ʒjv;

Spts. Menthol Pip. ad. q. s., - lbj.

Mix. Sig.—Use a teaspoonful in half a glass of tepid water as a mouth wash.

LOOSENING OF THE TEETH.—I have never seen a case of loosening of the teeth that did not have its beginning at the gingival margins as a result of infection or traumatism, excepting only cases of septic poisoning from apical infection from necrotic pulps.—Harlan, in *Dental Review*.

ARISTOL AS A PULP VARNISH.—Aristol dissolved in chloroform makes a good antiseptic and anodyne application to exposed pulps; it is adhesive and by the quick evaporation of the chloroform the resinous aristol is deposited as a uniform mechanical protector.—S. B. Luckie, in *Dental Cosmos*.

TO QUICKEN TACTILE IMPRESSIONS. — Dr. W. J. Younger suggests that the handles of scalers used in pyorrhea be coated with sealing wax at the portion of the handle where power is to be applied. This enables the operator to apply the instruments more definitely, as he gets a better appreciation of conditions at the working point.

IS THE ANTRUM EVER COMPLETELY DIVIDED BY A PARTITION WALL?—In cutting perhaps six hundred skulls I have failed to find an antrum that was completely divided by either bony or membranous septa. A bony or membranous septum may be found, but there is always a communication through it.—Dr. Cryer, in *International Journal*.

Filling as a Means of Providing Immunity.—We do not yet know how to manage caries otherwise than by so filling the teeth as to guard against its recurrence. We should always prepare for an emergency. Hence, I would suggest great care in cavity preparation, especially in cutting well toward the areas of immunity. And when I say this I do not necessarily mean we should cut only toward the gingival margins, so much, as toward the bucco-gingival and linguo-gingival in molars and bicuspids, squaring out that particular portion of the cavity. It is not generally so necessary that we cut the central part of the cavity farther toward the gingival. Often it is necessary to do so, but more frequently we should square those angles to the gum line and make the cavity box-form. It will be simpler to fill and is a form which the case generally demands. When we have done this the areas of liability have been removed. This particular region is not one of special liability when it is covered by *healthy* gum tissue. Then if the contact points are carefully made and properly guarded, recurrence at the central portion of the gingival margin is not likely to take place. If the contact points are not skillfully made they invite disease-producing conditions of the inter-dental tissues and recurrence of caries.—G. V. Black, *Dental Review*.

Setting Crowns with Gutta-Percha— When the stage of final fixing is reached and crown and pin dried, the pin is barbed as thoroughly as possible to afford a still better attachment for the gutta-percha, then the crown and pin are warmed and a suitable piece of ordinary pink base plate gutta-percha is heated quite hot in a pair of pliers over a Bunsen burner or alcohol lamp and immersed at once for a second in oil of eucalyptus, after which it is carefully formed around the pin and over the base of the crown and so trimmed as to leave but little more than will be required to fully fill and protect the root.

With this done the root face and canal are dried with a solution of bichlorid of mercury in ether by the aid of hot air, after which oil of eucalyptus is freely applied in the canal and on the base of the root with a cone of bibulous paper or cotton on a broach. The root is now ready for the crown, which is heated as hot as can be held comfortably in the fingers, when it can be pressed up to place most easily owing to the lubricating effect of the eucalyptus.

It is essential to note, that at this stage, heat is *not* applied direct to the gutta-percha, but that it is softened by the heat conducted to it by the crown and pin which retain the heat sufficiently long to give plenty of time to get the crown fully to place.

Removing the surplus, which should always exist, with suitable instruments, aided by eucalyptus, completes the operation.—Dr. L. J. Mitchell, in *Dental Review*.

